# Alzheimer's Disease: A Step Towards Prognosis Using Smart Wearables ^†^

**DOI:** 10.3390/ecsa-5-05742

**Published:** 2018-11-14

**Authors:** Antonella D. Pontoriero, Peter H. Charlton, Jordi Alastruey

**Affiliations:** Department of Biomedical Engineering, School of Biomedical Engineering and Imaging Sciences, King's College London, King's Health Partners, St Thomas' Hospital, London SE1 7EH, UK

**Keywords:** Alzheimer's, pulse wave, blood pressure, photoplethysmogram, hypoperfusion

## Abstract

Alzheimer's disease (AD) is the most common cause of dementia. Several haemodynamic risk factors for AD have been identified, including ageing, increased arterial stiffness, high systolic blood pressure (BP) and brain hypoperfusion. We propose a novel approach for assessing haemodynamic risk factors by analysing arterial pulse waves (PWs). The aim of this feasibility study was to determine whether features extracted from PWs measured by wearable sensors might have utility for stratifying patients at risk of AD. A numerical model of PW propagation was used to simulate PWs for virtual subjects of each age decade from 25 to 75 years (16 subjects in total), with subjects at each age exhibiting normal variation in arterial stiffness. Several PW features were extracted, and their relationships with AD risk factors were investigated. PWs at the wrist were found to exhibit changes with age and arterial stiffness, indicating that it may be possible to identify changes in risk factors from smart wearables. Several candidate PW features were identified which changed significantly with age for future testing. This study demonstrates the potential feasibility of assessing haemodynamic risk factors for AD from non-invasive PWs. These factors could be assessed from the PPG PW, which can be acquired by smart watches and phones. If the findings are replicated in clinical studies, then this may provide opportunities for patients to assess their own risk and make lifestyle changes accordingly.

## 1 Introduction

Alzheimer's disease (AD) is the most common cause of dementia, impacting patient well-being and healthcare costs. It is a progressive neurodegenerative brain disorder that disrupts normal brain structure and function, for which no cure is available. Its symptoms include memory loss, cognitive decline and a reduced ability to perform everyday tasks [1]. It is estimated that over 47 million people worldwide have AD, most of whom are over 65 years old [2]. Furthermore, it is predicted that the prevalence of AD will continue to rise dramatically over the coming decades with the increase in the number of people aged over 65 [2]. The cost of care for a patient with AD is often over €24,000 per annum, and can be as high as €70,000 [3]. Strategies for tackling AD may help to reduce the adverse impacts of AD on patients and resource utilisation.

It has been estimated that one third of AD cases might be attributable to modifiable risk factors [4]. Elevated blood pressure has been found to increase the risk of later-life cognitive impairment and AD [5]. Increased arterial stiffness has also been found to be predictive of cognitive decline [6]. It has also been observed that cerebral blood flow is reduced in AD, although it is not clear whether the link is causal [7]. Blood pressure and arterial stiffness increase with age, whilst cerebral blood flow decreases with age, which may contribute to the increased incidence of AD with age. Several lifestyle and therapeutic interventions have been proposed to modify these risk factors, and therefore potentially reduce the risk of AD [2].

It may be possible to use wearable sensors to assess an individual's risk of AD. Consumer devices such as smart watches and fitness bands routinely measure the arterial pulse wave (PW) [8], which is influenced by both the heart and blood vessels. Techniques have been proposed for analysing the PW to derive indicators of blood pressure, arterial stiffness, and the influence of ageing on the cardiovascular system. Based on this, we hypothesised that markers indicative of the risk of AD could be derived from PWs acquired from wearable sensors. Such markers could be reported to the individual, alongside recommendations of lifestyle changes, so that they could lower their risk of AD.

The aim of this feasibility study was to determine whether markers extracted from PWs might have utility for stratifying patients at risk of AD. Firstly, we investigated how changes in risk factors for AD (age, blood pressure and arterial stiffness) affect cerebral haemodynamics. Secondly, we assessed the potential utility of a range of features measured from the PW for detecting the changes in PW shape which are associated with risk factors for AD. This was achieved by analysing simulated PWs representative of subjects of different ages, and with different blood pressures and levels of arterial stiffness. The results indicate that risk factors for AD influence cerebral haemodynamics even during middle age, and that the accompanying changes in systemic haemodynamics could be detected using wearable sensors. If the findings are replicated in clinical studies, then this novel approach could provide early warning to patients at risk of AD, allowing them to adjust their lifestyle accordingly and potentially improve their cerebral haemodynamics.

## 2 Materials and Methods

### 2.1 Simulating Arterial Pulse Waves (PWs)

**Figure 1:**
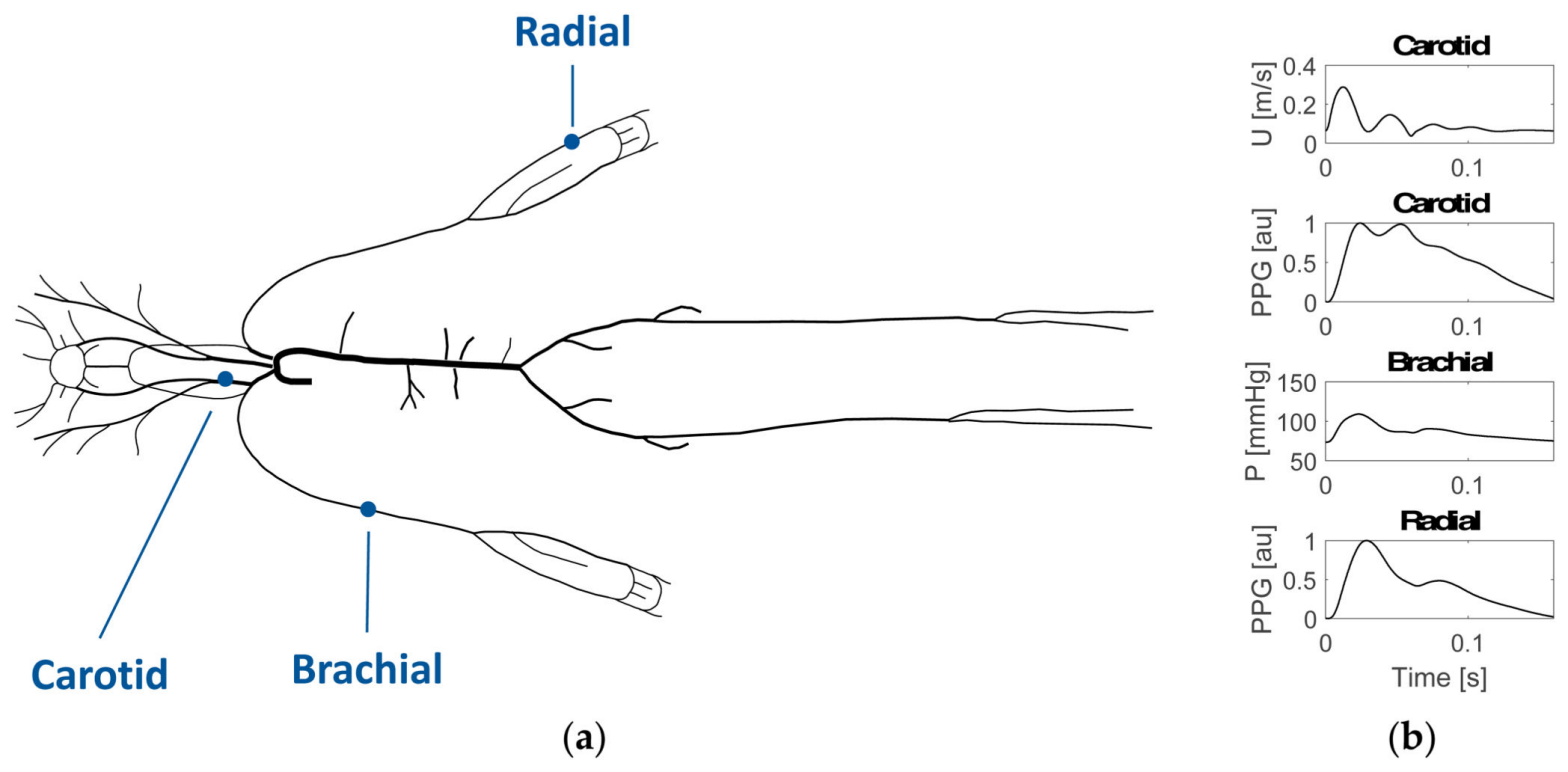
The model of arterial pulse wave (PW) propagation used to simulate PWs (**a**) the arterial network, showing the locations of the PWs analysed in this study (adapted from [11]); (**b**) the PWs for the 25-year old baseline subject at the internal carotid (flow velocity—U and photoplethysmogram—PPG), brachial (blood pressure—BP) and radial (PPG) sites.

A one-dimensional model of arterial PW propagation was used to simulate PWs at multiple arterial sites. The model is based on the physical principles of conservation of mass, linear momentum and energy [9]. The model generated pressure, flow and photoplethysmogram (PPG) PWs, as described in [10]. PPG PWs are representative of those measured by smart wearables using photoplethysmography, a measure of the volume of arterial blood in a tissue bed. It is characterized by inflow, outflow and junction matching boundary conditions. The arterial network, shown in Figure 1a, consisted of 116 arterial segments making up the larger arteries of the head, limbs, thoracic and abdominal organs [11]. Assumptions taken included: laminar flow, incompressible and Newtonian blood, and no energy losses at bifurcations. The pulse waves which were analysed in this study are shown in Figure 1b, namely the flow wave at the internal carotid artery, BP wave at the brachial artery and PPG waves at the neck (internal carotid artery) and wrist (radial artery).

PWs representative of a sample of healthy adults were simulated by adjusting the model input parameters to reflect changes in age and normal physiological variation. Firstly, PWs were simulated for subjects aged 25 to 75 (in 10-year intervals), following the approach in [10]. This involved changing the cardiac, arterial and vascular bed parameters specified to the model to simulate the typical changes in heart function, arterial stiffness and geometry, and blood pressure which occur with age. Secondly, at each age, PWs were simulated for a range of arterial stiffnesses indicative of typical inter-subject variation in arterial stiffness at that age. These simulations allowed the influences of AD risk factors on PWs to be investigated.

### 2.2 Investigating the Impact of Risk Factors on Haemodynamics

The impact of risk factors on blood pressure was assessed by calculating the mean, systolic and diastolic BPs at the brachial artery, representing the BP which is often measured in clinical settings and AD studies using a sphygmomanometer with a brachial cuff. Simulated changes in arterial stiffness were assessed by measuring the pulse wave velocity over the aortic path length, a technique which is commonly used to assess arterial stiffness. The impact on cerebral perfusion was assessed using the mean blood flow at the internal carotid artery. The internal carotid artery supplies blood to the circle of Willis, which in turn supplies blood to the brain.

### 2.3 Identifying Pulse Wave (PW) Features Indicative of Risk of Alzheimer's Disease (AD)

**Figure 2:**
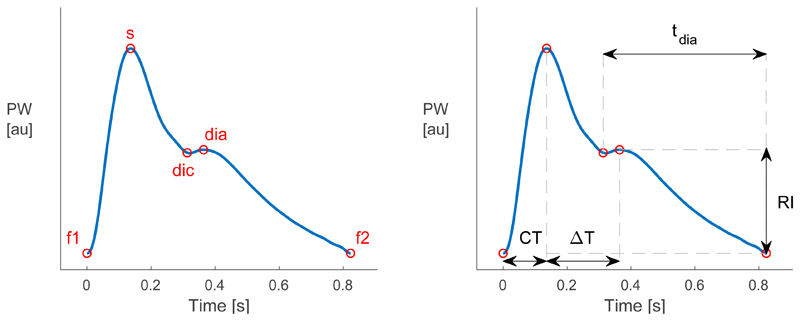
Extraction of features from photoplethysmogram (PPG) pulse waves (PWs), shown for the wrist PPG PW (**left**) Fiducial points were identified; (**right**) Feature measurements were extracted from the positions of fiducial points. Exemplary features are shown; the full list is provided in [11].

A total of 31 features were extracted from PPG PWs at the wrist (radial artery) and neck (carotid artery) to assess the potential utility of PPG measurements at these sites for assessing the risk of AD. The features, and the methods used to extract them, are described in [11]. Briefly, the features were extracted by identifying fiducial points on the PWs, and then extracting feature measurements from the positions of the fiducial points, as shown in Figure 2.

The suitability of each feature for identifying haemodynamic changes associated with increased risk of AD was assessed by comparing the features to those for the 25-year old subject. To do so, we used the data corresponding to 25- to 75-year-olds with elevated arterial stiffness. A one sample student's t-test was used to test whether the features extracted from the PWs for the 35- to 75-year-olds differed significantly from the value for the 25-year-old.

## 3 Results and Discussion

### 3.1 Changes in Risk Factors for Alzheimer's Disease (AD)

**Figure 3:**
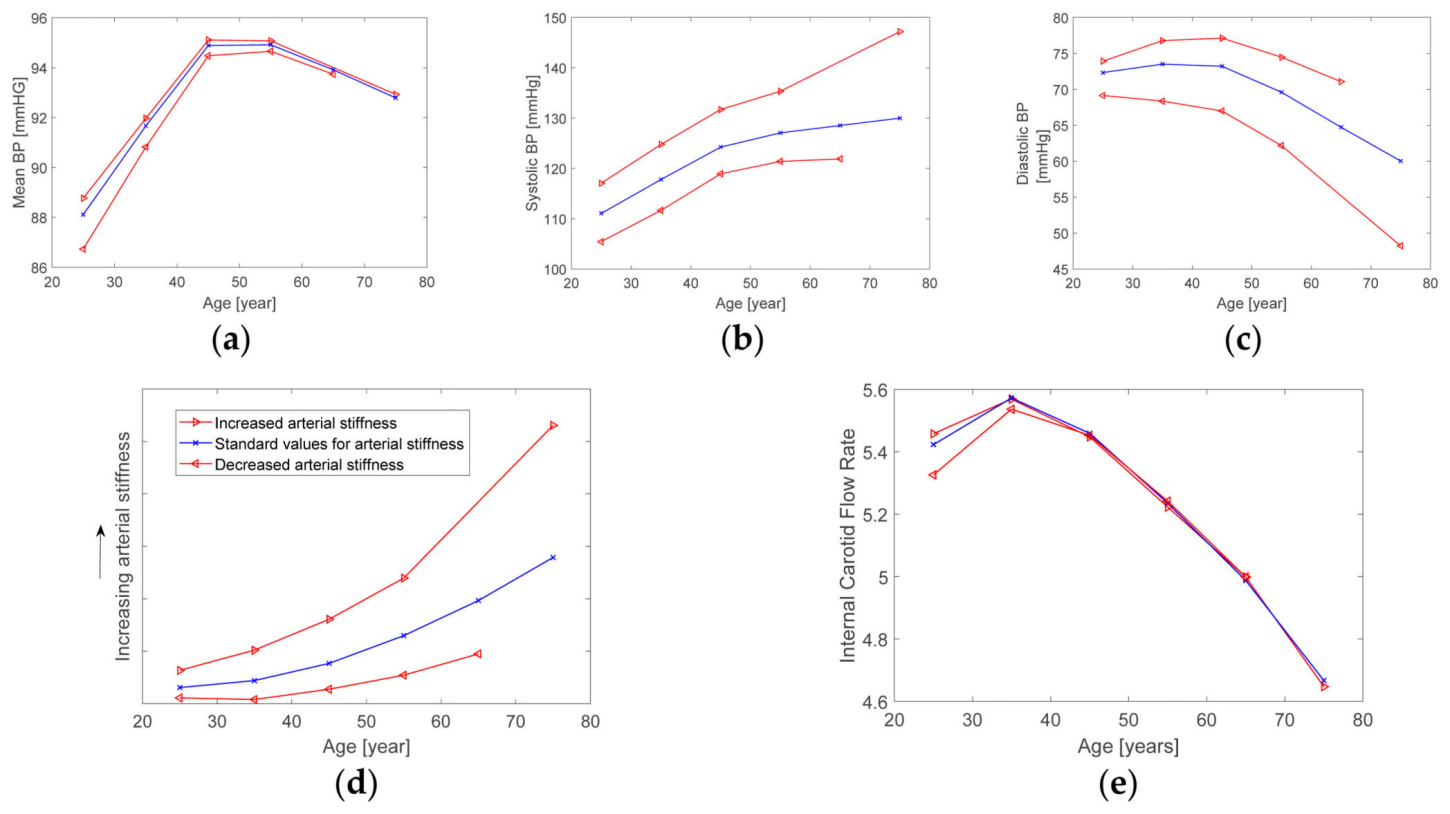
The simulated changes in risk factors for Alzheimer's Disease (AD). Changes in mean, systolic and diastolic blood pressure (BP) with age are shown in (**a–c**) respectively. The central blue lines indicate normal values for arterial stiffness at each age, upper red lines indicate increased stiffness, and lower red lines indicate lower stiffness. Reference arterial stiffness values are shown in (**d**), calculated from aortic pulse wave velocity. The changes in flow rate at the carotid artery are shown in (**e**).

The simulated changes in risk factors for AD are shown in Figure 3. The changes in BP (shown in (a), (b) and (c)) are similar to those observed *in vivo,* exhibiting increases in systolic and pulse pressure with age. Reference arterial stiffness values (shown in (d)) increased with age as reported in *in vivo* studies. These plots indicate that it may be possible to change an individual's risk factors for AD by treating elevated systolic BP and arterial stiffness. For instance, a 45-year-old with an elevated arterial stiffness (shown by the upper red line in (d)) has a similar arterial stiffness to that of a normal 65-year-old. Therefore, if interventions could reduce this arterial stiffness back to normal, then this could reduce the effective vascular age by approximately 20 years. The changes in carotid flow rate are shown in Figure 3e. There was a substantial reduction with age, but little change with arterial stiffness. This indicates that the increased incidence of AD in older subjects may in part be due to a decrease in brain perfusion with age. This was largely due to the reduction in cardiac output prescribed to the model with increasing age in this study. This indicates that increased cardiac output may help to avoid the reduction in cerebral perfusion with age, although interventions to achieve this may in turn increase BP, which could be detrimental.

### 3.2 Towards Pulse Wave Markers of Risk of Alzheimer's Disease (AD)

**Figure 4:**
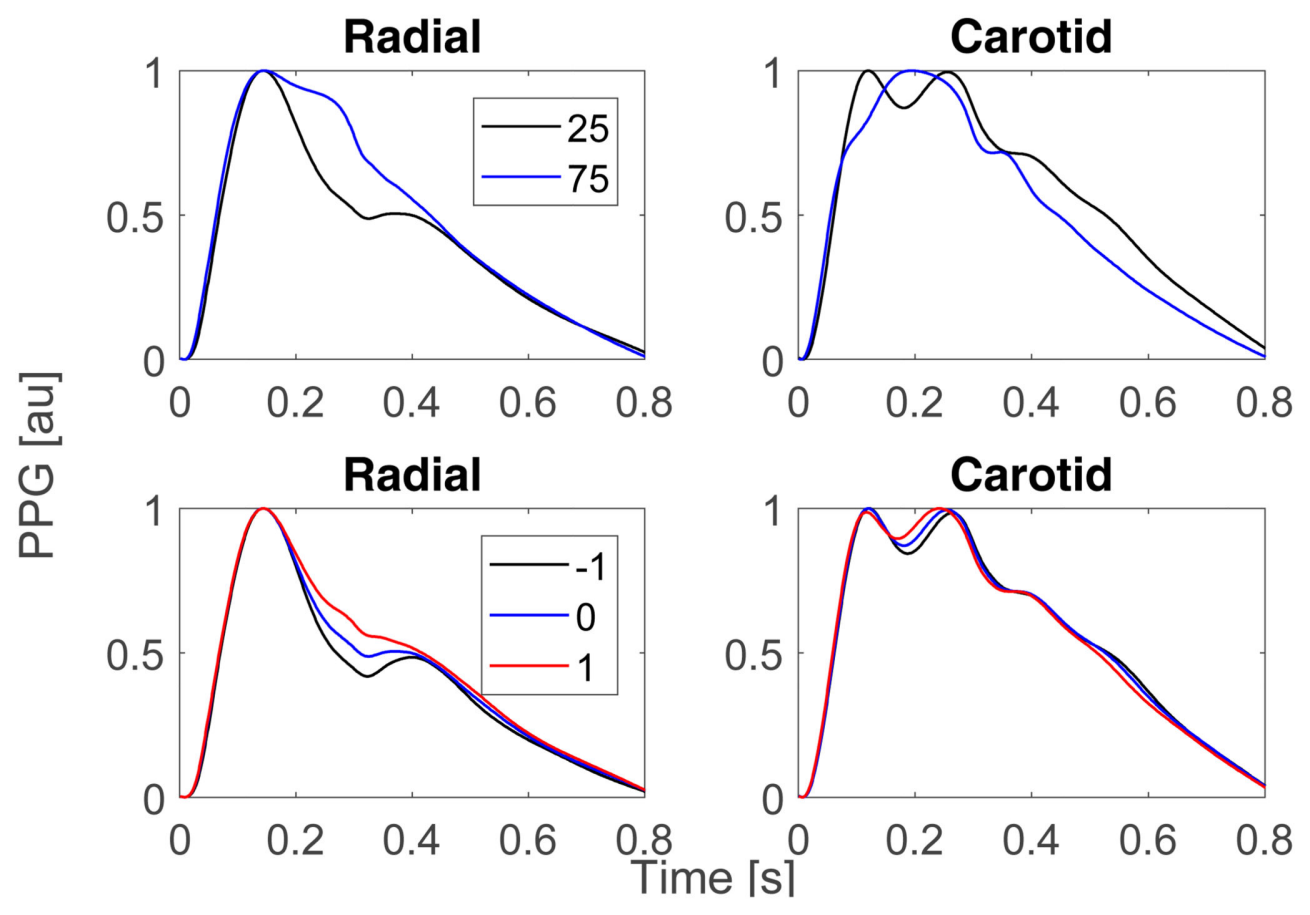
Photoplethysmogram (PPG) pulse waves (PWs) at the wrist (radial artery, **left**) and neck (carotid artery, **right**) sites, shown for subjects aged 25 and 75 (**upper** panels) and for different levels of arterial stiffness (**lower** panels). The **lower** plots show the PWs obtained when varying the arterial stiffness by ±1 standard deviation from typical values.

Figure 4 shows the effects of changes in age and arterial stiffness on PPG PWs. The upper panels show PPG PWs at the radial and carotid arteries for 25- and 75-year old subjects, showing changes with age. Firstly, the diastolic peak, at 0.4 s in the 25-year PWs, diminishes with age. Secondly, the relative strength of the secondary systolic peak, at 0.27 s, increases with age. Similar changes are observed in the PPG PWs for increased and decreased arterial stiffness in the lower panels of Figure 4. The change in radial PPG PWs is dominated by an increase in the strength of the secondary systolic peak with arterial stiffness, which results in the diastolic peak disappearing. The secondary systolic peak in the carotid PPG PWs occurs slightly earlier with increased stiffness. These marked changes with age and arterial stiffness suggest that it may be possible to identify changes in risk factors for AD from PWs. Indeed, a total of 28 out of the 31 features extracted from the PPG PW at the wrist changed significantly with increasing age, and 27 at the carotid artery. One would expect to observe similar changes when measuring PPG PWs using consumer devices: a smartwatch PPG measurement at the wrist, or the use of a smartphone to measure the PPG at the neck. Both of these approaches are suitable for use by the general population, providing opportunities to monitor markers of AD risk in everyday living, rather than just clinical settings.

### 3.3 Relevance for Prognosis

A wide range of risk factors for AD have been identified [2]. It is believed that cerebral haemodynamics, and in particular brain hypoperfusion [7,12–14], are associated with development of AD. The hypothesis, of brain hypoperfusion as a cause for AD, originates from studies that relate general anesthesia to dementia [15] and to brain blood flow reduction [16]. The approach presented here is designed for detecting systemic changes associated with causal cerebral changes. Remarkably, hypoperfusion seems to be correlated to Parkinson's disease [17] and other forms of dementia [18]. Therefore, this approach may have wider applications beyond AD.

### 3.4 Limitations and Future Work

The key limitation of this study is that it was conducted using a model of pulse wave propagation rather than *in vivo* data. This approach can be used to analyse the influence of individual factors on PWs, such as arterial stiffness, and to elucidate mechanisms relating systemic haemodynamics to those which are difficult to measure *in vivo,* such as cerebral blood flow. Further testing is required with wearable sensor data to determine whether these findings are replicated *in vivo.* In the future it would be informative to conduct a control-case longitudinal study based on measuring brain perfusion. Suitable cohorts include: healthy patients, AD patients and patients with some symptoms but no diagnosis of AD, such as those with a positive cognitive test result.

## 4 Conclusions

In this study, a model of blood flow was used to investigate the influence of risk factors for AD on PWs. We found that the PPG PW at the wrist is affected by changes in age and arterial stiffness. This suggests that it may be possible to use wearable sensors such as smart watches to provide early warning of increased risk of AD due to haemodynamic risk factors. If the findings are replicated in clinical studies then this approach could be used to encourage patients to adjust their lifestyle and receive treatments to lower their risk of AD.
